# A Novel Arithmetic Optimization PDR Algorithm for Smartphones

**DOI:** 10.3390/s25237129

**Published:** 2025-11-21

**Authors:** Mingze Zhang, Aigong Xu

**Affiliations:** School of Geomatics, Liaoning Technical University, Fuxin 123000, China; 47211051@stu.lntu.edu.cn

**Keywords:** AO-PDR, smartphones, fitness function, optimal system parameters, heading correction mechanism, location-based service

## Abstract

In order to accurately and reasonably set the Pedestrian Dead Reckoning (PDR) system parameters, a novel arithmetic optimization PDR algorithm (AO-PDR) for smartphones is proposed. Firstly, the AO-PDR sets system parameters such as the binary threshold, sliding window size, step length estimation coefficient, and motion state judgment threshold. Based on the positioning error, step deviation, and step length deviation the fitness function of Arithmetic Optimization Algorithm (AOA) is established. Secondly, throughout the initial exploration and development stages, the AOA efficiently searches for the minimum fitness and obtains the optimal system parameters, which are then applied to step detection, step length estimation, and heading correction to solve the pedestrian gait, step length, and heading. Based on the pedestrian motion state, the heading correction mechanism is established. Finally, the pedestrian coordinates are calculated based on the step length and heading. In order to comprehensively evaluate the performance of AO-PDR, four experimenters walked around two experimental sites with three smartphones, respectively, and collected 24 sets of data. The parameter optimization and pedestrian positioning experiments were designed. The experimental results show that AO-PDR can obtain the optimal parameters efficiently and accurately. The mean optimal fitness is 1.352, and the mean running time is 164.85 s. The AO-PDR has high adaptability, efficiency, and stability for different pedestrians and smartphones. The mean positioning error is 0.2893 m, and the standard deviation of positioning error is 0.341 m, which meets the accuracy requirements of pedestrian location-based services.

## 1. Introduction

Location-based service (LBS) has always played an important role in people’s daily lives. Among them, the Global Navigation Satellite System (GNSS) has provided all-weather, high-precision navigation information for global users, and greatly changed people’s lifestyles since it was initially proposed in the 1970s [1,2]. Nevertheless, buildings are becoming taller and taller, and indoor and underground spaces are becoming increasingly abundant. In large indoor and shaded environments, GNSS cannot be effectively positioned [3]. At the same time, some studies have pointed out that people spend 70% to 90% of their time indoors, and the demand for indoor LBS is strong [4,5]. Therefore, the accurate and reliable indoor positioning technology is the current research hotspot of LBS.

BLE [6,7], UWB [8,9,10] and Wi-Fi [11,12,13] have high accuracy in indoor positioning. However, in practical applications, the above indoor positioning technologies require a pre-built database in order to function. Thus, it is difficult to implement accurate positioning indoors without existing infrastructure. The cost of this is high. Visual Grounding (VG) [14,15,16], Geomagnetic Matching (GM) [17,18,19] and Pedestrian Dead Reckoning (PDR) [20,21,22] have strong autonomy. Pedestrian positioning can be completed only by using image, geomagnetic field or inertial information. However, VG is susceptible to light, and its image-matching algorithms are computationally intensive. Similarly, GM is affected by metal equipment, and the mismatches are more serious. In contrast, with the continuous advancements of Micro-Electro-Mechanical System (MEMS), smartphone-based PDR has emerged as the mainstream research of indoor positioning [23,24]. PDR can output high-precision position information in a short time, but due to the low accuracy of smartphone built-in MEMS sensors, the positioning error would accumulate over time [25,26]. Consequently, building a stable and reliable PDR system is still the key focus of research on pedestrian indoor positioning.

The PDR needs to collect tri-axial acceleration and angular velocity during pedestrian movement. It then calculates gait, step length, and heading through step detection, step length estimation, and heading correction, ultimately computing pedestrian coordinates [27,28,29]. To enhance the positioning performance of PDR, some scholars have proposed step detection algorithms such as peak detection [30], Finite State Machine (FSM) [31], and Fast Fourier Transform (FFT) [32] by using the periodic variation in the tri-axial acceleration and angular velocity. In addition, some scholars have proposed nonlinear step length estimation algorithms, such as the Weinberg model, the Scarlet model, and the Kim model, using the physical relationship between step length and acceleration [30,33]. At the same time, other scholars have proposed Kalman Filter (KF) [34], Extended Kalman Filter (EKF) [35], Unscented Kalman Filter (UKF) [36], Particle Filter (PF) [37], and Heuristic Drift Elimination (HDE) [38] by using tri-axial acceleration, angular velocity, and geomagnetic field. In the above algorithms, the parameters are generally set as fixed empirical values or determined by a large number of experiments. However, experimental factors such as pedestrians and smartphones will significantly affect the parameter setting. As a result, reasonable and accurate setting of system parameters can improve the positioning accuracy of PDR.

In order to set the PDR system parameters reasonably and accurately, this paper proposes a novel arithmetic optimization PDR algorithm (AO-PDR) for smartphone. The contributions of this paper are summarized as follows: (1) to establish a comprehensive optimization mechanism, six system parameters—binary threshold, sliding window size, step length estimation coefficient and motion state judgment threshold—were set to cover the three stages of PDR. Based on the positioning error, step deviation and step length deviation, the fitness function of Arithmetic Optimization Algorithm (AOA) was established. (2) Through the initial, exploration and development stages, the multiplication and division operator were used to enhance the dispersion of global search, and then the addition and subtraction operator were used to enhance the accuracy of local development. The AOA can effectively balance the global search and local development capabilities and efficiently obtains the optimal system parameters. (3) The optimal system parameters were applied to step detection, step length estimation, and heading correction. The binary threshold was used to detect pedestrian gait information, and a sliding window was established to eliminate false steps. Furthermore, the Weinberg model was used to estimate the pedestrian step length. Based on quaternion, the heading was solved to judge the pedestrian motion state, and the heading correction mechanism was established. Finally, pedestrian coordinates were calculated using step length and heading.

The remainder of the paper is organized as follows: Section 1 introduces the smartphone-based PDR system, including step detection, step length estimation and heading correction. In Section 2, the novel smartphone-based AO-PDR is introduced. The specific process of system parameter and AOA optimization is introduced in detail. Section 3 introduces the experimental design and analyzes the experimental results. We present the conclusions and reveal some potential research topics for the future in Section 4.

## 2. The Smartphone-Based PDR System

**Figure 1:**
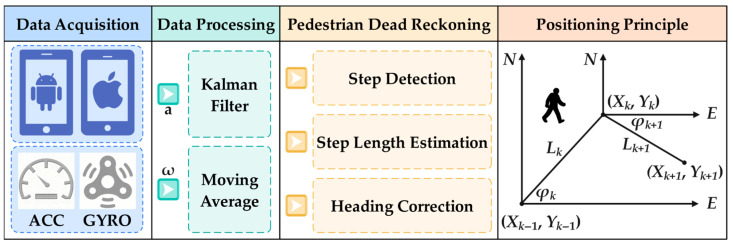
The positioning process of PDR system.

With the rapid innovation and development of MEMS, the smartphone-based PDR system has emerged as the mainstream research of indoor positioning. This system uses the smartphone built-in MEMS sensors to collect the acceleration and angular velocity. Based on this, the step length and heading are estimated to calculate the pedestrian coordinates. The positioning process of PDR system is shown in Figure 1.

Supposing that the position coordinates of step k−1 are $\left(X_{k-1},Y_{k-1}\right)$, the step length and heading of step k are $L_{k}$ and $\varphi_{k}$, respectively. Then, the position coordinates of step k are calculated as follows:

(1) $$\left\{\begin{array}{c} X_{k}=X_{k-1}+L_{k}\sin(\varphi_{k})\\ Y_{k}=Y_{k-1}+L_{k}\cos(\varphi_{k}) \end{array}\right.$$

### 2.1. Step Detection and Step-Length Estimation

**Figure 2:**
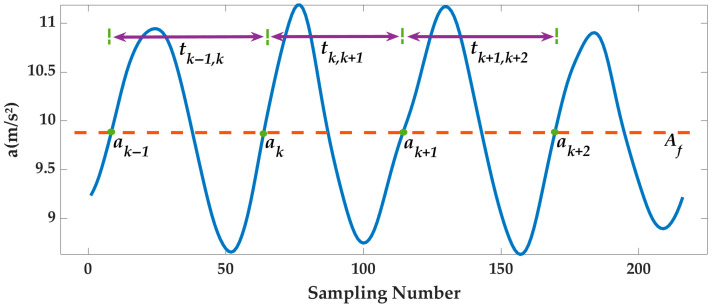
Variation characteristics of resultant acceleration.

As the first stage of PDR system, step detection provides gait information for step length estimation and heading correction, which affects the accuracy of step length and heading. At present, most step detection algorithms detect gait information based on the variation characteristics of resultant acceleration. During movement, the pedestrians’ legs are highly symmetrical, and the velocity and position change regularly with the center of gravity. A gait period is defined as the period from heel-off to foot-flat. Each gait period includes acceleration and deceleration stages, as shown in Figure 2. At the beginning of the gait period, the pedestrian lifts the foot and the resultant acceleration gradually increases. When the foot is raised to the highest point, the resultant acceleration is at its largest. Then, the lifted foot falls, and the resultant acceleration decreases. Subsequently, the pedestrian enters the next gait period, and the acceleration stage and the deceleration stage will alternate periodically.

Therefore, there is always a binary threshold $A_{f}$ to reasonably divide each gait period into the acceleration and deceleration stages. Using $A_{f}$ to judge the resultant acceleration $a_{k}$, a binary state array State is obtained as follows:

(2) $$State=\left\{\begin{array}{l} 1,\;a_{k}>A_{f}\\ 0,\;a_{k}\leq A_{f} \end{array}\right.$$

where State = 1 indicates that the gait period is in the acceleration stage, and State = 0 indicates that the gait period is in the deceleration stage.

On this basis, it is judged whether the time interval $t_{k-1,k}$ of the gait period meets the pedestrian motion characteristics. If the time interval $t_{k-1,k}$ is too long or too short, the gait period will be considered as false step. To this end, a backward sliding window of size w is established, and the binary state array State is optimized by mean smoothing and rounding, Then, a new binary state array $State^{\prime }$ is obtained as follows:

(3) $$State^{\prime }=\mathrm{round}\left[\left(State_{i}+State_{i-1}+State_{i-2}+\cdots +State_{i-w+1}\right)/w\right]$$

where round represents the rounding function.

**Figure 3:**
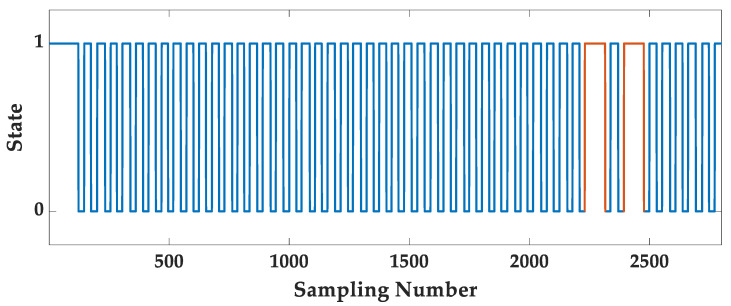
The binary state.

Figure 3 shows an example of the binary state array $State^{\prime }$.

As shown in Figure 3, there are still a small number of false steps. Therefore, the temporary variable m is set to count the number of binary states “1”, and the false steps are further detected and eliminated. The m is counted as follows:

(4) $$m=\left\{\begin{array}{ll} m+1, & State^{\prime }=1\\ 0, & State^{\prime }=0\;\&\;m<w/2\\ m+1, & State^{\prime }=0\;\&\;m\geq w/2\; \end{array}\right.$$

when m > w, the current gait period detection is completed. Gait information such as the start/end time and resultant acceleration of the current gait period are recorded.

The Weinberg model estimates the step length based on the nonlinear relationship between step length and resultant acceleration. The model is established as:

(5) $$L=K\cdot \sqrt[{4}]{a_{\max}-a_{\min}}$$

where $a_{\max}$ and $a_{\min}$ are the maximum and minimum values of the resultant acceleration in a gait period, respectively, and K is the step length estimation coefficient.

### 2.2. Heading Correction

Step 1: Heading solution.

The direction cosine, Euler angle, and quaternion are often used to update the attitude. Among them, the quaternion requires few calculations and is easy to implement. Therefore, in this paper, we use it the quaternion to update the pedestrian attitude and calculate the heading. The quaternion ${\left[q_{0}\;q_{1}\;q_{2}\;q_{3}\right]}^{T}$ is updated as follows:

(6) $${\left[\begin{array}{c} q_{0}\\ q_{1}\\ q_{2}\\ q_{3} \end{array}\right]}_{k+1}={\left[\begin{array}{c} q_{0}\\ q_{1}\\ q_{2}\\ q_{3} \end{array}\right]}_{k}+\frac{dt}{2}{\left[\begin{array}{cccc} 0 & -\omega_{x} & -\omega_{y} & -\omega_{z}\\ \omega_{x} & 0 & \omega_{z} & -\omega_{y}\\ \omega_{y} & -\omega_{z} & 0 & \omega_{x}\\ \omega_{z} & \omega_{y} & -\omega_{x} & 0 \end{array}\right]}_{k}{\left[\begin{array}{c} q_{0}\\ q_{1}\\ q_{2}\\ q_{3} \end{array}\right]}_{k}$$

where ${\left[\cdot \right]}_{k}$ represents the information at time k, ${\left[\omega_{x}\;\omega_{y}\;\omega_{z}\right]}^{T}$ represents the tri-axial angular velocity, and dt is the sampling period, dt = 0.01.

The heading $\varphi_{k}$ at time k is calculated as follows:

(7) $$\varphi_{k}=arctan\frac{2(q_{1}q_{2}-q_{0}q_{3})}{q_{0}^{2}-q_{1}^{2}+q_{2}^{2}-q_{3}^{2}}$$

Eight main headings are set, and the offset $E_{k}$ of the heading $\varphi_{k}$ relative to the main headings is calculate as follows:

(8) $$E_{k}=\frac{\Delta }{2}-mod\left(\varphi_{k},\Delta \right)$$

where mod represents modulus function, and Δ represents main heading interval, Δ = 45°.

When $E_{k}$ > 0, it shows that the heading $\varphi_{k}$ is left compared with the main heading, and a correction $c_{k}$ needs to be added. On the contrary, it shows that the heading $\varphi_{k}$ is right compared with the main heading, and a correction $c_{k}$ needs to be subtracted. Therefore, the signum function $\mathrm{sgn}\left(E_{k}\right)$ can be constructed as follows:

(9) $$\mathrm{sgn}\left(E_{k}\right)=\left\{\begin{array}{c} 1,\;E_{k}>0\\ 0,\;E_{k}=0\\ -1,\;E_{k}<0 \end{array}\right.$$

The heading $\varphi_{k}$ at time k is corrected as follow:

(10) $$\varphi_{k}=\varphi_{k}+\mathrm{sgn}\left(E_{k}\right)\times c_{k}$$

where $c_{k}$ is related to the performance of the smartphone built-in gyroscope.

Step 2: Motion state judgment.

When pedestrians move or turn along the non-main heading, the heading is prone to overcorrection. Therefore, based on the continuous headings $\varphi_{k-2}$, $\varphi_{k-1}$ and $\varphi_{k}$, the pedestrian motion state is determined to establish the heading correction mechanism. The first two steps of the pedestrian are identified as moving straight along the main heading, and then the heading is judged from the third step.

Straight/turning: To determine whether the continuous headings $\varphi_{k-2}$, $\varphi_{k-1}$, and $\varphi_{k}$ meet the threshold, the judgment conditions are as follows:

(11) $$\left\{\begin{array}{c} \;J_{1}=(\varphi_{k}-\varphi_{k-1})\times (\varphi_{k-1}-\varphi_{k-2})\\ J_{2}=\left|\varphi_{k}-\varphi_{k-1}\right|\;\\ J_{3}=\left|\varphi_{k}-\varphi_{k-1}\right|+\left|\varphi_{k-1}-\varphi_{k-2}\right| \end{array}\right.$$

(12) $$Z_{k}^{1}=\left\{\begin{array}{cc} 0, & J_{1}<0\;\&\;J_{2}<SJ_{1}||J_{3}<SJ_{2}\\ 1, & otherwise \end{array}\right.$$

where $J_{1}$, $J_{2}$, and $J_{3}$ are three different judgment conditions of the continuous heading, $SJ_{1}$ and $SJ_{2}$ are the thresholds for judging the straight/turning.

When $Z_{k}^{1}$ = 0, the pedestrian walks straight ahead and keeps the correction $c_{k}$. When $Z_{k}^{1}$ = 1, the pedestrian turns and adjusts the correction $c_{k}$.

The main heading/the non-main heading: The mean heading of three continuous steps is mapped to the interval [0, Δ], and then the angle between the mean heading and the main heading is calculated. The judgment conditions are as follows:

(13) $${\overset{⌢}{\varphi }}_{k}=mod\left(\left(\varphi_{k}+\varphi_{k-1}+\varphi_{k-2}\right)/3,\Delta \right)$$

(14) $$Z_{k}^{2}=\left\{\begin{array}{cc} 0, & min\left({\overset{⌢}{\varphi }}_{k},\Delta /2-{\overset{⌢}{\varphi }}_{k}\right)<MJ\\ 1, & otherwise\; \end{array}\right.$$

where ${\overset{⌢}{\varphi }}_{k}$ is the mean heading of continuous heading, MJ is the threshold for judging the main heading/non-main heading, min is the minimum function.

When $Z_{k}^{1}$ = 0, the pedestrian moves along the main heading and keeps the correction $c_{k}$. When $Z_{k}^{1}$ = 1, the pedestrian moves along the non-main heading and adjusts the correction $c_{k}$.

Step 3: Heading correction mechanism.

When $Z_{k}^{1}$ =1 || $Z_{k}^{2}$ = 1, the difference $\Delta \varphi_{k}$ between the heading $\varphi_{k}$ and the mean heading of $\varphi_{k}$, $\varphi_{k-1}$, $\varphi_{k-2}$ is calculated as follows:

(15) $$\Delta \varphi_{k}=\varphi_{k}-\frac{\varphi_{k}+\varphi_{k-1}+\varphi_{k-2}}{3}$$

A size 5 sliding window is established to calculate the variance of the difference $\Delta \varphi_{k}$, and the correction ${c_{k}}^{\prime }$ is dynamically adjusted as follows:

(16) $${c_{k}}^{\prime }=\frac{5c_{k}}{\sum_{i=n-4}^{n}{\left(\Delta \varphi_{k}-\Delta \bar{\varphi }\right)}^{2}}$$

where $\Delta \bar{\varphi }$ is the mean heading of $\Delta \varphi_{k}$ in the sliding window.

The heading $\varphi_{k}$ at time k is corrected as follows:

(17) $$\varphi_{k}=\varphi_{k}+\mathrm{sgn}\left(E_{k}\right)\times {c_{k}}^{\prime }$$

when $Z_{k}^{1}$ = 0 || $Z_{k-1}^{1}$ = 1, the heading $\varphi_{k}$ is corrected as follows:

(18) $$\varphi_{k+m}=\{\begin{array}{cc} \varphi_{k+m}-{\overset{⌢}{\varphi }}_{k}+\Delta , & \Delta \varphi_{k}>\Delta /2\\ \varphi_{k+m}-{\overset{⌢}{\varphi }}_{k}, & otherwise \end{array}$$

where m = 0, 1, 2…end, that is, all the current and future headings are corrected.

## 3. The Smartphone-Based AO-PDR System

The smartphone-based PDR system sets the binary threshold $A_{f}$ and the sliding window size w in the step detection stage. In the step length estimation stage, the coefficient K is set. In the heading correction stage, the straight judgment thresholds $SJ_{1}$, $SJ_{2}$ and the main heading judgment threshold MJ are set. The PDR system parameters are shown in Table 1. Due to the differences in pedestrians and smartphones, the system parameters need to be continuously optimized to obtain more accurate positioning results. However, manual optimization of parameters requires extensive experiments and experience, with subjective bias, and it is difficult to obtain the global optimal parameters.

**Figure 4:**
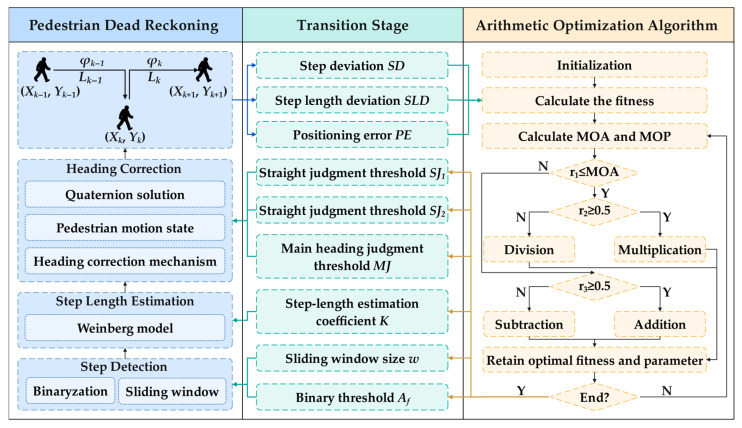
AO-PDR system framework.

Therefore, this paper introduces AOA into the smartphone-based PDR system and establishes a novel arithmetic optimization PDR system (AO-PDR). The system framework is shown in Figure 4.

The AOA is a metaheuristic optimization algorithm which has been proposed by Faramarzi et al. in 2020 [39]. The algorithm performs global search and local development according to the distribution characteristics of operators “addition, subtraction, multiplication and division”. The multiplication and division operator are used to enhance the dispersion of global search, and then the addition and subtraction operator are used to enhance the accuracy of local development. Consequently, the AOA can prevent the optimization from falling into local optimal parameters. It also reduces the time and cost of manual optimization and efficiently obtains the global optimal parameters.

The AOA is divided into the initial, exploration, and development stages. The adaptive transformation between the exploration and development stages can help AOA efficiently search for the optimal parameters. The diversity of potential parameters is maintained, enabling a wider search. The specific steps are as follows:

Step 1: The initial stage.

Define the fitness function: the positioning error PE, step deviation SD and step length deviation SLD are calculated as:

(19) $$PE=\frac{\sum_{k=1}^{n}\sqrt{{\left(X_{k}-x_{k}\right)}^{2}+{\left(Y_{k}-y_{k}\right)}^{2}}}{n}$$

(20) $$SD=\left|\frac{RealStep-Step}{RealStep}\right|$$

(21) $$SLD=\frac{\sum_{k=1}^{n}\left|RealL_{k}-L_{k}\right|}{n}$$

where $\left(X_{k},Y_{k}\right)$ represent the real pedestrian coordinates, $\left(x_{k},y_{k}\right)$ represent the solution pedestrian coordinates, RealStep represents the real step number, Step represents the solution step number, $RealL_{k}$ represents the real step length, and $L_{k}$ represents the solution step length.

In order to comprehensively establish the optimization mechanism, based on the positioning error PE, step deviation SD and step length deviation SLD, the fitness function Fit is established as follows:

(22) Fit=PE+SD+SLD

Define the math optimizer accelerated (MOA): MOA updates the control coefficients of the exploration stage and the development stage, dynamically adjusts the step length and direction of the optimization, and focuses the candidate parameters on a more accurate area. The MOA is calculated as follows:

(23) $$MOA=Min+CurI\times \left(\frac{Max-Min}{MaxI}\right)$$

where Max and Min represents the maximum and minimum values of MOA, respectively, CurI represents the current number of iterations, and MaxI represents the maximum number of iterations.

Define the math optimizer probability (MOP): MOP avoids over-reliance on specific operators by controlling the usage frequency of different operators. The effects of different operators are balanced to improve the stability of optimization. The MOP is calculated as follows:

(24) $$MOP=1-\frac{CurI^{1/\alpha }}{MaxI^{1/\alpha }}$$

where α represents the effective accuracy of the iterative process, α = 5.

Step 2: The exploration and development stages.

$r_{1}$, $r_{2}$ and $r_{3}$ are random numbers between [0, 1].

When $r_{1}\leq MOA$, the AOA enters the exploration stage. In this stage, AOA uses multiplication and division operators to carry out global search, improve the dispersion of system parameters, and realize global optimization. The exploration stage is written as follows:

(25) $$KT_{i,j}=\left\{\begin{array}{cc} B_{j}/\left(MOP+\varepsilon \right)\times \left(\left(ub-lb\right)\times \mu +lb\right), & r_{2}<0.5\\ B_{j}\times MOP\times \left(\left(ub-lb\right)\times \mu +lb\right), & r_{2}\geq 0.5 \end{array}\right.$$

where $B_{j}$ represents the location of the current optimal parameter, and ε represents the minimum constant.

When $r_{1}>MOA$, the AOA enters the development stage. In this stage, AOA uses addition and subtraction operators to reduce the dispersion of parameters, improve the local development ability, and approach the optimal parameters faster. The development stage is written as follows:

(26) $$KF_{i,j}=\left\{\begin{array}{c} B_{j}-MOP\times \left(\left(ub-lb\right)\times \mu +lb\right),\;r_{3}<0.5\\ B_{j}+MOP\times \left(\left(ub-lb\right)\times \mu +lb\right),\;r_{3}\geq 0.5 \end{array}\right.$$

In summary, the pseudo-code of smartphone-based AO-PDR system is shown in Algorithm 1.
**Algorithm 1:** Smartphone-based AO-PDR systemInputs: a, ω, t, N, n, MaxI, α, μ, μb, lb
**AOA system**     **While**
CurI < MaxI
**do**        Calculate Fit, MOA, and MOP by Equations (19)–(24)**    Exploration phase**        **If**$\;r_{2}<0.5$
**then**            
$KT_{i,j}=B_{j}/\left(MOP+\varepsilon \right)\times \left(\left(ub-lb\right)\times \mu +lb\right)$

        **Else**
            
$KT_{i,j}=B_{j}*MOP\times \left(\left(ub-lb\right)\times \mu +lb\right)$

        **End if**

    **Exploitation phase**
        **If** $r_{3}<0.5$
**then**
            $KF_{i,j}=B_{j}-MOP\times \left(\left(ub-lb\right)\times \mu +lb\right)$

        **Else**
            
$KF_{i,j}=B_{j}+MOP\times \left(\left(ub-lb\right)\times \mu +lb\right)$

        **End if**
**PDR system**    Update $A_{f}$, w, K, $SJ_{1}$, $SJ_{2}$, MJ
    Calculate State, $L_{k}$, $\varphi_{k}$ by Equations (2)–(10)    **For**
i := 3 **To** num **do**        Calculate $Z_{k}^{1}$, $Z_{k}^{2}$ by Equations (11) and (14)    **If**
$Z_{k}^{1}$ = 1 *||*
$Z_{k}^{2}$ = 1 **do**        
$\varphi_{k}=\varphi_{k}+\mathrm{sgn}\left(E_{k}\right)\times \frac{5c_{k}}{\sum_{k=n-4}^{n}{\left(\Delta \varphi_{k}-\Delta \bar{\varphi }\right)}^{2}}$

    **else**
$\varphi_{k+m}=\left\{\begin{array}{cc} \varphi_{k+m}-\Delta \varphi_{k}+\Delta , & \Delta \varphi_{k}>\Delta /2\\ \varphi_{k+m}-\Delta \varphi_{k} & otherwise \end{array}\right.$
    **End if**

    **End for**
    Update X, Y by Equation (1)Output: L, φ, X, Y


## 4. Experimental Verification and Analysis

**Figure 5:**
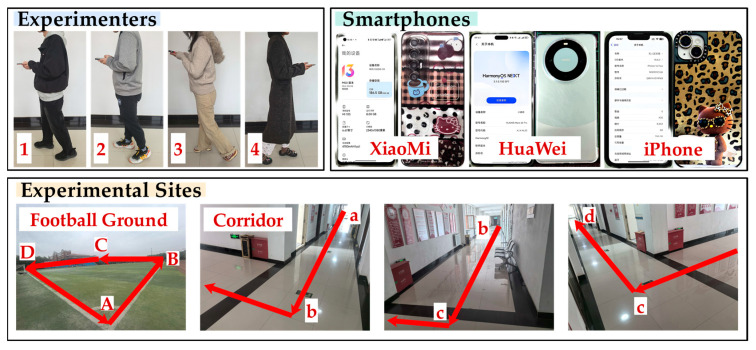
The graphical representation of experimental factors.

In order to comprehensively verify and analyze the performance of the AO-PDR, this study sets up a parameter optimization experiment and a pedestrian positioning experiment. Four experimenters walked around two experimental sites while holding three smartphones in their hands and collected acceleration and angular velocity at 100 HZ, as shown in Figure 5. The whole collection process was continuous without pause when turning the corner. There were two males and two females, and their specific information is shown in Table 2. The three smartphones used were Xiaomi 10S, Huawei Mate 60 Pro and iPhone 14 Plus, and their specific information is shown in Table 3. The two experimental sites were an outdoor football ground and an indoor corridor. The football ground (A→B→C→D) was a closed rectangle with 105 m × 68 m. The indoor corridor (a→b→c→d) was composed of three corridors, measuring 39 m, 50.6 m and 46.2 m, respectively. The dataset “SmartPhone-AccGyroData-PDR” contains 24 sets of data and has been shared on Github (https://github.com/Wx10101/Smartphone-AccGyroData-PDR (accessed on 22 October 2025)).

### 4.1. Parameter Optimization Experiment

In order to comprehensively verify and analyze the parameter optimization ability of the AO-PDR, Bayesian Optimization (BO) [40], Bald Eagle Search (BES) [41] and Equilibrium Optimizer (EO) [39] are selected as comparative algorithms. BO is an automatic tuning algorithm proposed by J.Snoek in 2012, BES is a metaheuristic algorithm proposed by Faramarzi in 2020, and EO is a swarm intelligence algorithm proposed by Alsatter in 2020. For the AOA and three comparison algorithms, the fitness function is established based on the positioning error, step deviation, and step length deviation, as shown in Equations (19)–(22).

**Figure 6:**
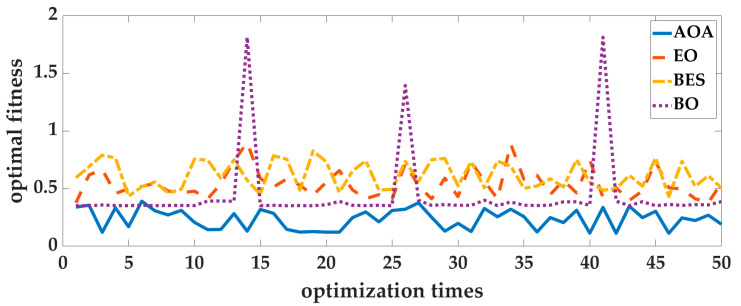
The optimal fitness of 50 optimizations.

**Figure 7:**
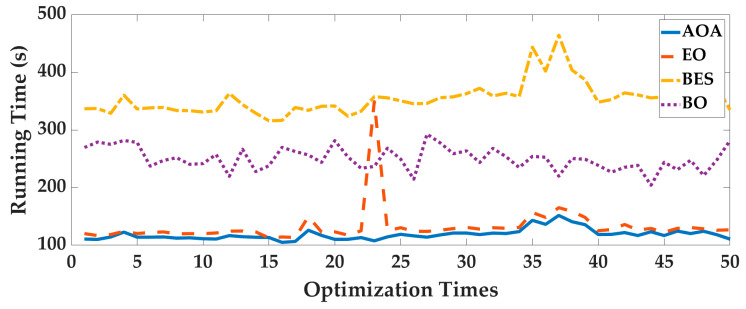
The running time of 50 optimizations.

**Figure 8:**
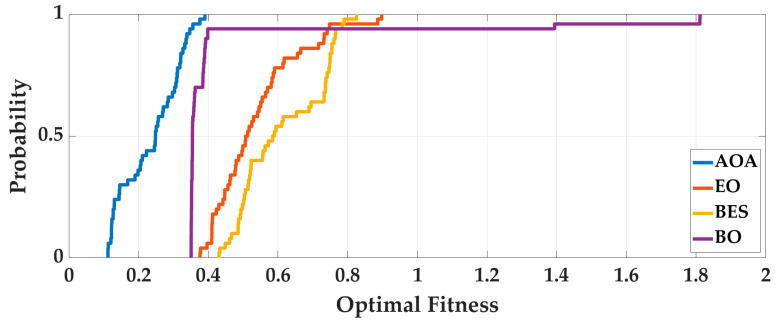
The CDF of optimal fitness of 50 optimizations.

**Figure 9:**
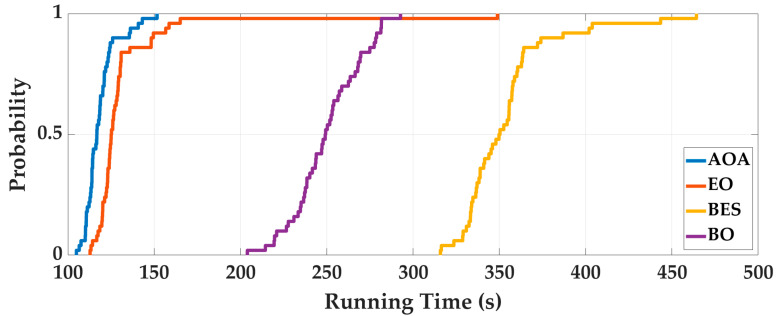
The CDF of running time of 50 optimizations.

Case 1: In order to eliminate the contingency of AOA optimization, the four intelligent optimization algorithms are optimized 50 times repeatedly. Taking the Experimenter 3 flat-end Huawei Mate 60 Pro walking in the corridor as an example, we analyze the optimization results. The optimal fitness is shown in Figure 6, and the running time is shown in Figure 7. To deeply analyze the optimal fitness and running time, CDFs are shown in Figure 8 and Figure 9, and then the mean value, standard deviation, minimum value and maximum value are shown in Table 4.

From Figure 6, Figure 7, Figure 8 and Figure 9 and Table 4, for 50 optimizations, the mean value and standard deviation of optimal fitness of AOA are 0.2354 and 0.0858, and then the mean value and standard deviation of the running time of AOA are 118.61 s and 9.28 s. This shows that the AOA has high efficiency and stability. Compared with BO, BES and EO, the AOA has advantages in terms of parameter optimization and running time. The mean value of optimal fitness is reduced by 0.2064, 0.3788, and 0.3023, and then the mean value of the running time is reduced by 131.64 s, 234.95 s, and 13.76 s.

**Figure 10:**
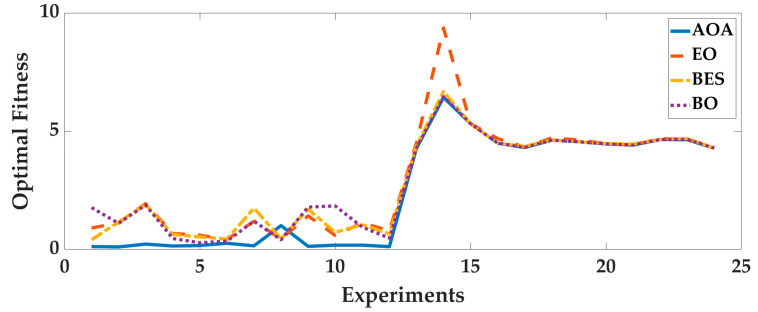
The optimal fitness of 24 sets of data.

**Figure 11:**
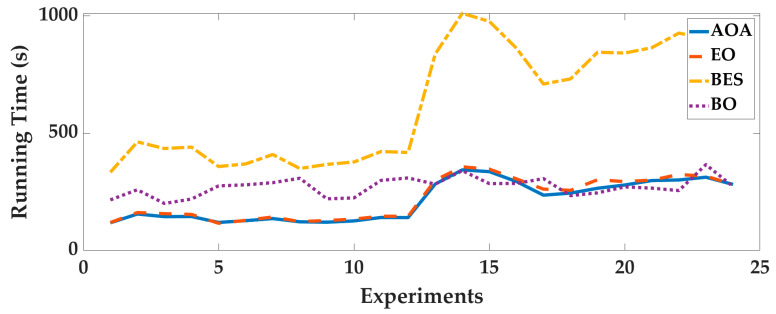
The running time of 24 sets of data.

**Figure 12:**
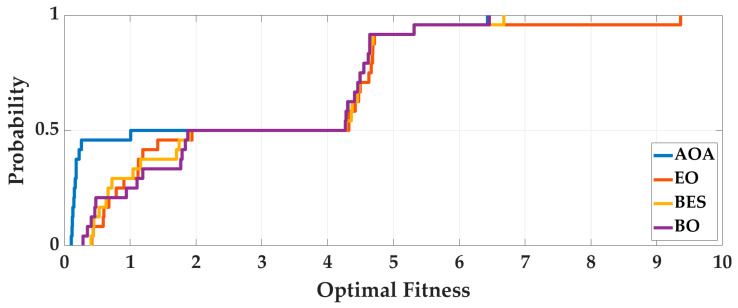
The CDF of optimal fitness of 24 sets of data.

**Figure 13:**
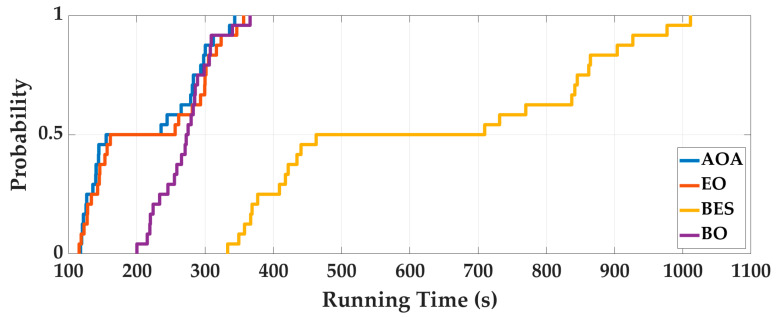
The CDF of running time of 24 sets of data.

Case 2: In order to analyze the adaptability of AOA to different pedestrians and smartphones, four intelligent optimization algorithms were used to optimize the 24 sets of data, we analyze the optimization results. The optimal fitness is shown in Figure 10, and the running time is shown in Figure 11. To deeply analyze the optimal fitness and running time, CDFs are shown in Figure 12 and Figure 13, and the mean value, standard deviation, minimum value and maximum value are shown in Table 5.

From Figure 10, Figure 11, Figure 12 and Figure 13 and Table 5, for different pedestrians and smartphones, the mean value and standard deviation of optimal fitness of AOA are 2.4685 and 2.3277, and then the mean value and standard deviation of running time of AOA are 211.09 s and 83.58 s. This shows that the AOA has high adaptability, efficiency and stability. Compared with BO, BES and EO, the AOA has advantages in terms of parameter optimization and running time. The mean value of optimal fitness is reduced by 0.4047, 0.3801 and 0.5034, and then the mean value of running time is reduced by 60.09 s, 414.9 s, and 9.56 s.

### 4.2. Pedestrian Positioning Experiment

In order to comprehensively verify and analyze the pedestrian positioning ability of the AO-PDR, Fixed Correction-PDR (FC-PDR) and Fixed Parameter-PDR (FP-PDR) are selected as comparative algorithms, and the specific information is shown in Table 6. The step detection and step length estimation of three algorithms are based on binary detection and the Weinberg model, as shown in Equations (2)–(5). The parameters of AO-PDR are obtained by the AOA, and the parameters of comparison algorithms are set according to the references and experience.

**Figure 14:**
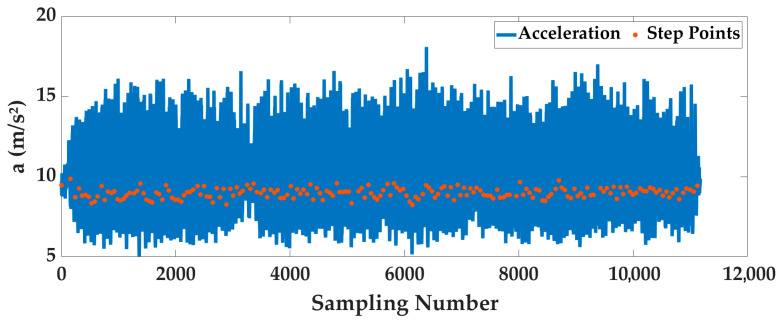
The pedestrian gait information.

**Figure 15:**
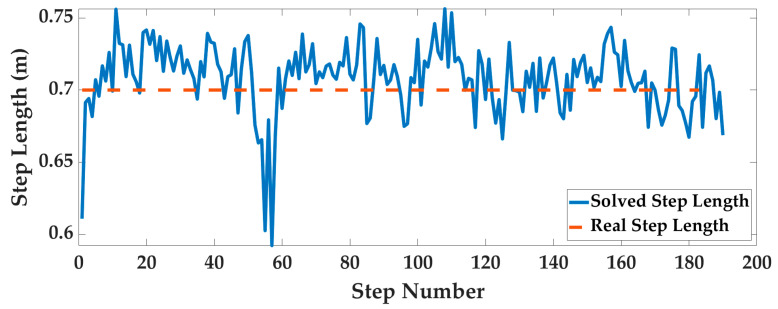
The pedestrian step length information.

**Figure 16:**
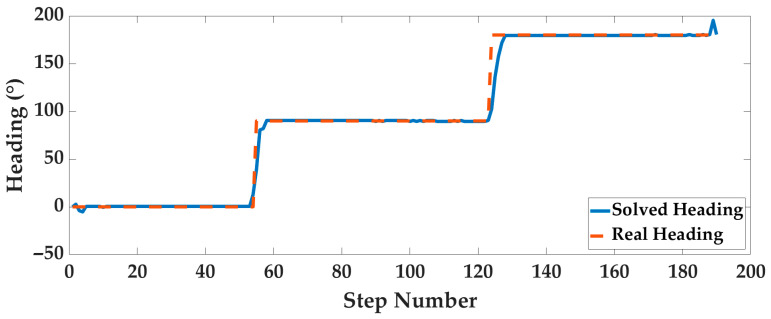
The pedestrian heading information.

Case3: Taking Experimenter 3 flat-end Huawei Mate 60 Pro walking in the corridor as an example, the pedestrian positioning results are displayed. The optimal system parameters are obtained by the AOA and then input into the PDR system to calculate the gait, step length, and heading, as shown in Figure 14, Figure 15 and Figure 16.

**Figure 17:**
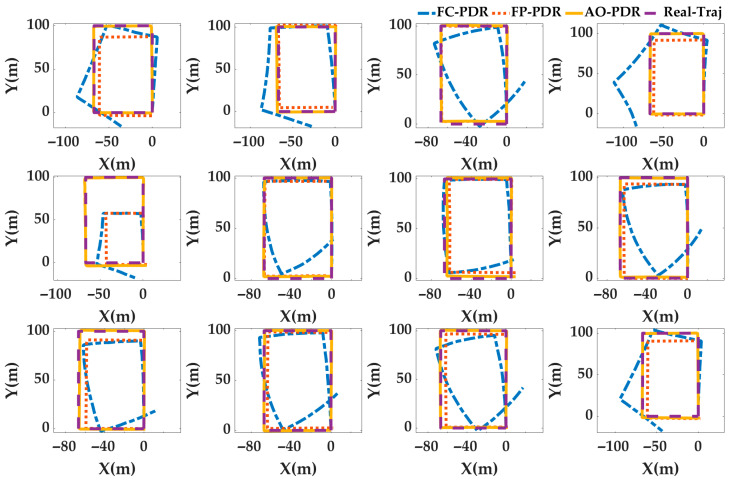
Pedestrian trajectory on the football ground.

**Figure 18:**
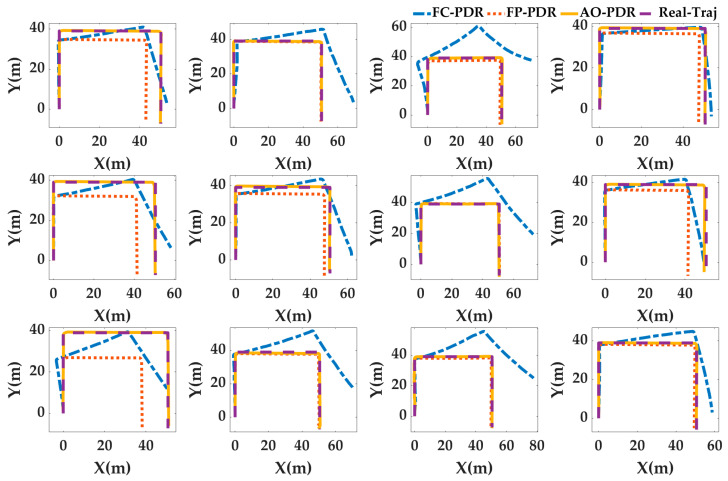
Pedestrian trajectory in the corridor.

**Figure 19:**
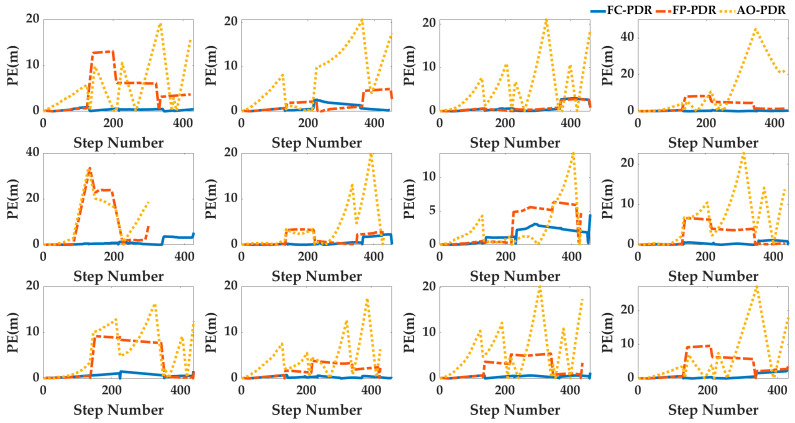
Positioning error of pedestrian trajectory on the football ground.

**Figure 20:**
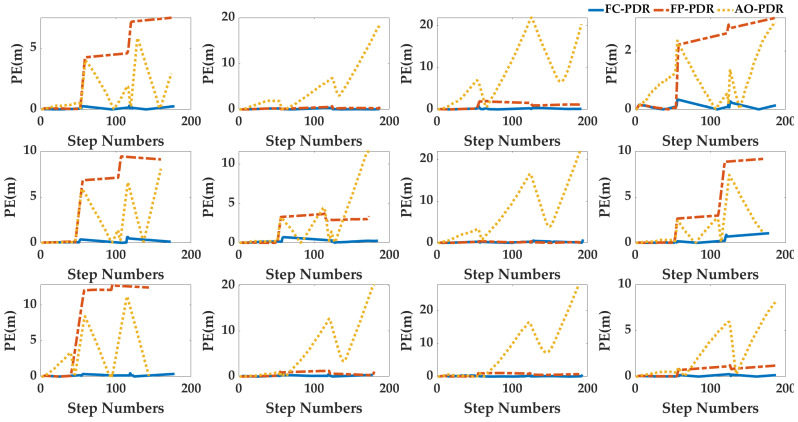
Positioning error of pedestrian trajectory in the corridor.

**Figure 21:**
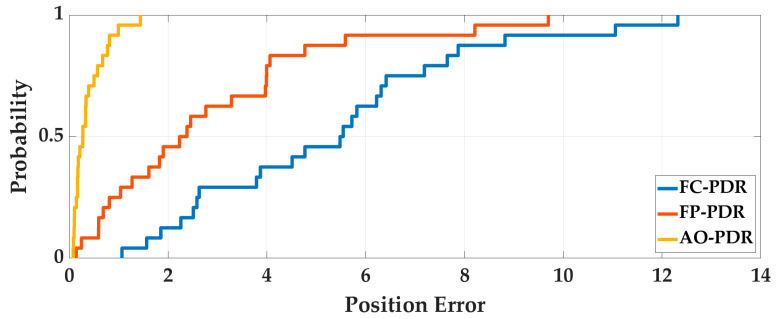
The CDF of positioning error.

Case4: In order to analyze the adaptability of AO-PDR to different pedestrians and smartphones, three PDR systems were used to locate the 24 sets of data. The pedestrian positioning results were analyzed, and the pedestrian trajectories are shown in Figure 17 and Figure 18, and the positioning errors are shown in Figure 19 and Figure 20. To deeply analyze the positioning error, the CDF is shown in Figure 21, and the mean value, standard deviation, minimum value and maximum value are shown in Table 7.

From Figure 17, Figure 18, Figure 19, Figure 20 and Figure 21 and Table 7, for different pedestrians and smartphones, the mean value, standard deviation, minimum value, and maximum value of positioning error of AO-PDR are 0.3864 m, 0.341 m, 0.0821 m and 1.4416 m, respectively. This shows that the AO-PDR has high adaptability, efficiency, and stability. Compared with FC-PDR and FP-PDR, the AO-ODR has advantages in pedestrian positioning. The mean value of positioning error was reduced by 4.9411 m and 2.4539 m, and the standard deviation of positioning error was reduced by 2.5676 m and 2.0855 m.

## 5. Conclusions

In order to accurately and reasonably set the PDR system parameters and improve pedestrian positioning accuracy and adaptability, a novel arithmetic optimization PDR algorithm (AO-PDR) for smartphones is proposed. The AO-PDR uses the positioning error, step deviation, and step length deviation to establish the fitness function, and efficiently obtain the optimal system parameters such as binary threshold, sliding window size, step length estimation coefficient, and motion state judgment threshold. The optimal parameters are applied to step detection, step length estimation and heading correction to calculate pedestrian coordinates. In order to comprehensively evaluate the performance of AO-PDR, a parameter optimization experiment and a pedestrian positioning experiment are designed. (1) For the parameter optimization experiment, AOA is better than BO, BES, and EO. The mean value of optimal fitness was reduced by 0.3056, 0.3795, and 0.4029, and then the mean value of running time was reduced by 95.87 s, 324.93 s and 11.66 s. (2) For the pedestrian positioning experiment, AO-PDR is better than FC-PDR and FP-PDR. The mean value of positioning error was reduced by 4.9411 m and 2.4539 m, and then the standard deviation of positioning error was reduced by 2.5676 m and 2.0855 m. (3) On the whole, the AO-PDR can obtain the optimal parameters efficiently and accurately. The mean value of optimal fitness was 1.352, and the mean value of running time was 164.85 s. The AO-PDR has high adaptability, efficiency, and stability for different pedestrians and smartphones. The mean value of positioning error was 0.2893 m, and the standard deviation of positioning error was 0.341 m, which meets the accuracy requirements of pedestrian location-based services. In the future, we plan to add geomagnetic and visual features to further improve the accuracy and adaptability of the AO-PDR.

## Figures and Tables

**Table 1:** The settings of the system parameters.

Parameters	Stages	Values	Meanings
$A_{f}$	Step Detection	∈[9.6, 10.4]	Binary threshold
w		∈[40, 60]	Sliding window size
K	Step Length Estimation	∈[0.3, 0.8]	Step length estimation coefficient
$SJ_{1}$	Heading Correction	∈[13, 17]	Straight judgment threshold 1
$SJ_{2}$		∈[8, 12]	Straight judgment threshold 2
MJ		∈[8, 12]	Main heading judgment threshold

**Table 2:** Specific information of four experimenters.

Information	Experimenter1	Experimenter2	Experimenter3	Experimenter4
Gender	Male	Male	Female	Female
Height (cm)	168	180	170	176
Weight (kg)	56	80	69	58

**Table 3:** Specific information of three smartphones.

Information	Xiaomi 10s	Huawei Mate 60 Pro	iPhone 14 Plus
Systems	MIUI 13.0.10	HarmonyOS Next 5.1.0	iOS 18.6.2
Internal storage	8G + 256G	12G + 512G	8G + 256G
Battery capacity	4780 mAh	5000 mAh	4323 mAh
Processors	Qualcomm Snapdragon 870	HiSilicon Kirin 9000S	Apple A15 Bionic
Accelerometers	lsm6dso Accelerometer Non-wakeup	rgm 3-axis Accelerometer	An accelerometer from Bosch Sensortec
Gyroscopes	lsm6dso Gyroscope Non-wakeup	rgm 3-axis Gyroscope	A gyroscope from Bosch Sensortec

**Table 4:** Technical indexes of optimal fitness and running time of 50 optimizations.

Technical Indexes	Optimal Fitness				Running Time (s)			
	BO	BES	EO	AOA	BO	BES	EO	AOA
Mean	0.4418	0.6142	0.5378	0.2354	250.25	353.56	132.37	118.61
Std	0.3182	0.1213	0.1240	0.0858	19.88	27.70	33.19	9.28
Min	0.3510	0.4309	0.3766	0.1122	204.01	315.87	112.87	104.95
Max	1.8115	0.8257	0.8980	0.3905	292.75	464.22	348.91	151.68

**Table 5:** Technical indexes of optimal fitness and running time of 24 sets of data.

Technical Indexes	Optimal Fitness				Running Time (s)			
	BO	BES	EO	AOA	BO	BES	EO	AOA
Mean	2.8732	2.8486	2.9719	2.4685	271.18	625.99	220.65	211.09
Std	1.9691	2.0288	2.3137	2.3277	40.47	245.73	87.90	83.58
Min	0.2851	0.4074	0.4194	0.1071	200.31	333.31	115.73	117.69
Max	6.4583	6.6778	9.3628	6.4303	366.12	1011.28	356.67	343.61

**Table 6:** The specific information of AO-PDR and comparison algorithms.

Algorithms	Step Detection	Step Length Estimation	Heading Correction	Parameters
**FC-** **PDR**	Binary detection with fixed parameters	Weinberg model with fixed parameter	The heading is solved by quaternion and corrected according to the correction.	[9.88, 50, 0.55, null, null, null]
**FP** **-PDR**	Binary detection with fixed parameters	Weinberg model with fixed parameter	Based on FC-PDR, the heading correction is adjusted according to the pedestrian motion state.	[9.88, 50, 0.55, 15, 10, 10]
**AO** **-PDR**	Binary detection with optimal parameters	Weinberg model with optimal parameter	Based on FP-PDR, the heading correction mechanism is established according to the motion state.	Obtained by the AOA

**Table 7:** Technical indexes of positioning error.

Technical Indexes	FC-PDR	FP-PDR	AO-PDR
Mean (m)	5.3275	2.8403	0.3864
Std (m)	2.9086	2.4265	0.3410
Min (m)	1.0683	0.1434	0.0821
Max (m)	12.3194	9.6978	1.4416

## Data Availability

The original contributions presented in the study are included in the article, further inquiries can be directed to the corresponding author.

## References

[1] Shu Y., Xu P., Niu X., Chen Q., Qiao L., Liu J. (2022). High-rate attitude determination of moving vehicles with GNSS: GPS, BDS, GLONASS, and Galileo. IEEE Trans. Instrum. Meas..

[2] Cheng S., Wang F., Li G., Geng J. (2023). Single-frequency multi-GNSS PPP-RTK for smartphone rapid centimeter-level positioning. IEEE Sens. J..

[3] Niu X., Liu T., Kuang J., Li Y. (2020). A novel position and orientation system for pedestrian indoor mobile mapping system. IEEE Sens. J..

[4] Zhou Z., Feng W., Li P., Liu Z., Xu X., Yao Y. (2023). A fusion method of pedestrian dead reckoning and pseudo indoor plan based on conditional random field. Measurement.

[5] Zhang L., Jiao K., He W., Wang X. (2024). Anchor deployment optimization for range-based indoor positioning systems in non-line-of-sight environment. IEEE Sens. J..

[6] You Y., Wu C. (2021). Hybrid indoor positioning system for pedestrians with swinging arms based on smartphone IMU and RSSI of BLE. IEEE Trans. Instrum. Meas..

[7] Dinh T.-M.T., Duong N.-S., Sandrasegaran K. (2020). Smartphone-based indoor positioning using BLE iBeacon and reliable lightweight fingerprint map. IEEE Sens. J..

[8] Zhang Y., Wang N., Weng S., Li M., Mou D., Han Y. (2020). Emergency Positioning Method of Indoor Pedestrian in Non-Cooperative Navigation Environment Based on Virtual Reference Node Array/INS. IEEE Sens. J..

[9] Zhu X., Yi J., Cheng J., He L. (2020). Adapted error map based mobile robot UWB indoor positioning. IEEE Trans. Instrum. Meas..

[10] Yang Y., Wang X., Li D., Chen D., Zhang Q. (2022). An improved indoor 3-D ultrawideband positioning method by particle swarm optimization algorithm. IEEE Trans. Instrum. Meas..

[11] Tao Y., Zhao L. (2018). A novel system for WiFi radio map automatic adaptation and indoor positioning. IEEE Trans. Veh. Technol..

[12] Feng X., Nguyen K.A., Luo Z. (2024). A wi-fi rss-rtt indoor positioning model based on dynamic model switching algorithm. IEEE J. Indoor Seamless Position Navig..

[13] Sun M., Wang Y., Zheng N., Chen G., Li Z., Bi J. (2025). Smartphone based indoor localization system using Wi-Fi RTT/Magnetic/PDR based on an improved particle filter. IEEE Trans. Instrum. Meas..

[14] Tang C., Sun W., Zhang X., Zheng J., Wu W., Sun J. (2023). A novel fingerprint positioning method applying vision-based definition for wifi-based localization. IEEE Sens. J..

[15] Yan J., He G., Basiri A., Hancock C. (2019). 3-D passive-vision-aided pedestrian dead reckoning for indoor positioning. IEEE Trans. Instrum. Meas..

[16] Jia S., Ma L., Yang S., Qin D. (2022). A novel visual indoor positioning method with efficient image deblurring. IEEE Trans. Mob. Comput..

[17] De Angelis G., Pasku V., De Angelis A., Dionigi M., Mongiardo M., Moschitta A., Carbone P. (2014). An indoor AC magnetic positioning system. IEEE Trans. Instrum. Meas..

[18] Yeh S.-C., Hsu W.-H., Lin W.-Y., Wu Y.-F. (2019). Study on an indoor positioning system using Earth’s magnetic field. IEEE Trans. Instrum. Meas..

[19] Kusche R., Schmidt S.O., Hellbrück H. (2021). Indoor positioning via artificial magnetic fields. IEEE Trans. Instrum. Meas..

[20] Lin F., Cai Q., Liu Y., Chen Y., Huang J., Peng H. (2024). Pedestrian dead reckoning method based on array imu. IEEE Sens. J..

[21] Shi L.-F., Zhao Y.-L., Liu G.-X., Chen S., Wang Y., Shi Y.-F. (2018). A robust pedestrian dead reckoning system using low-cost magnetic and inertial sensors. IEEE Trans. Instrum. Meas..

[22] Jiang C., Chen Y., Chen C., Jia J., Sun H., Wang T., Hyyppä J. (2022). Smartphone PDR/GNSS integration via factor graph optimization for pedestrian navigation. IEEE Trans. Instrum. Meas..

[23] Xia H., Zuo J., Liu S., Qiao Y. (2018). Indoor localization on smartphones using built-in sensors and map constraints. IEEE Trans. Instrum. Meas..

[24] Wang Q., Fu M., Wang J., Luo H., Sun L., Ma Z., Li W., Zhang C., Huang R., Li X. (2022). Recent advances in pedestrian inertial navigation based on smartphone: A review. IEEE Sens. J..

[25] Jin Z., Zhang X., Liu G., Guo M., Su Y., Lu M. (2025). Flexible gaits adaptive pedestrian dead reckoning system: Precision positioning across diverse gaits. IEEE Sens. J..

[26] Wu L., Guo S., Han L., Baris C.A. (2024). Indoor positioning method for pedestrian dead reckoning based on multi-source sensors. Measurement.

[27] Yao Y., Pan L., Fen W., Xu X., Liang X., Xu X. (2020). A robust step detection and stride length estimation for pedestrian dead reckoning using a smartphone. IEEE Sens. J..

[28] Li W., Chen R., Yu Y., Wu Y., Zhou H. (2021). Pedestrian dead reckoning with novel heading estimation under magnetic interference and multiple smartphone postures. Measurement.

[29] Harindranath A., Arora M. (2024). A systematic review of user-conducted calibration methods for MEMS-based IMUs. Measurement.

[30] Zhao G., Wang X., Zhao H., Jiang Z. (2023). An improved pedestrian dead reckoning algorithm based on smartphone built-in MEMS sensors. AEU-Int. J. Electron. Commun..

[31] Bi J., Zhen J., Yao G., Sang W., Ning Y., Guo Q. (2023). Improved Finite State Machine Step Detection Algorithm for Smartphone. Geomat. Inf. Sci. Wuhan Univ..

[32] Dirican A.C., Aksoy S. (2017). Step counting using smartphone accelerometer and fast Fourier transform. Sigma J. Eng. Nat. Sci..

[33] Kochka K.V., Evseev A.D., Chugunov A.A. Synthesis of the step detection and step length estimation algorithms based on imu measurements. Proceedings of the 2023 International Russian Automation Conference (RusAutoCon).

[34] Wu Q., Li Z., Shao K. (2023). Location accuracy indicator enhanced method based on MFM/PDR integration using Kalman filter for indoor positioning. IEEE Sens. J..

[35] Yamagishi S., Jing L. (2023). The Extended Kalman Filter with Reduced Computation Time for Pedestrian Dead Reckoning. IEEE Sens. Lett..

[36] Tong X., Su Y., Li Z., Si C., Han G., Ning J., Yang F. (2019). A double-step unscented Kalman filter and HMM-based zero-velocity update for pedestrian dead reckoning using MEMS sensors. IEEE Trans. Ind. Electron..

[37] Pei L., Liu D., Zou D., Choy R.L.F., Chen Y., He Z. (2018). Optimal heading estimation based multidimensional particle filter for pedestrian indoor positioning. IEEE Access.

[38] Zhang W., Wei D., Yuan H. (2020). The improved constraint methods for foot-mounted PDR system. IEEE Access.

[39] Faramarzi A., Heidarinejad M., Stephens B., Mirjalili S. (2020). Equilibrium optimizer: A novel optimization algorithm. Knowl.-Based Syst..

[40] Victoria A.H., Maragatham G. (2021). Automatic tuning of hyperparameters using Bayesian optimization. Evol. Syst..

[41] Alsattar H.A., Zaidan A.A., Zaidan B. (2020). Novel meta-heuristic bald eagle search optimization algorithm. Artif. Intell. Rev..

